# Central Versus Peripheral Invasive Arterial Blood Pressure Monitoring in Liver Transplant Surgery

**DOI:** 10.7759/cureus.33095

**Published:** 2022-12-29

**Authors:** Uoo R Kim, Annie T Wang, Samantha H Garvanovic, Simona I Lupu, Esther Gow-Lee, David A Sanner

**Affiliations:** 1 Anesthesiology and Perioperative Medicine, Loma Linda University School of Medicine, Loma Linda, USA; 2 Anesthesiology, Kaiser Permanente, Los Angeles, USA; 3 Anesthesiology, Loma Linda University Medical Center, Loma Linda, USA; 4 Anesthesiology, Massachusetts General Hospital, Boston, USA; 5 Anesthesiology, Loma Linda University School of Medicine, Loma Linda, USA

**Keywords:** vasopressors, peripheral arterial pressure, central arterial pressure, adult hemodynamics, hemodynamic monitoring, clinical liver transplantation

## Abstract

Introduction

Invasive blood pressure monitoring is essential in liver transplant surgery due to expected major hemodynamic shifts. The use of central versus peripheral arterial access, however, is institution-dependent, which can affect clinical decisions regarding vasopressor therapy. Although there are studies that demonstrate inconsistencies based on arterial cannulation sites, few studies have compared femoral and radial artery blood pressures in patients undergoing liver transplant surgery. To our knowledge, there are no studies investigating the differences between continuous minute-to-minute femoral and radial artery measurements during all three phases of liver transplant surgery.

Objective

The main objective of this study was to evaluate for any differences between central and peripheral blood pressure measurements in liver transplant surgery and to assess for any correlation between vasopressor infusion dose and femoral-arterial pressure differences.

Methods

In this retrospective study, we reviewed and studied the data of 61 patients with American Society of Anesthesiologists (ASA) grade 4 who underwent liver transplant surgery at Loma Linda University Medical Center between January and December of 2019. All patients had both femoral and radial arterial lines placed for liver transplant surgery. Femoral and radial arterial blood pressure values were obtained continuously over 60 minutes in the pre-anhepatic phase, 45 minutes during the anhepatic phase, and 60 minutes into the neo-hepatic phase. Vasopressor infusion doses were also recorded for each patient during these time frames.

Results

This pilot study found statistically significant differences between the mean femoral and radial systolic blood pressure (SBP; p < 0.0001), diastolic blood pressure (DBP; p < 0.0001), and mean arterial pressure (MAP; p < 0.0001) during all phases of liver transplantation. The meanSBP and MAP differences between femoral and radial arteries were highest (femoral blood pressure reading higher than radial blood pressure measurements) in the late anhepatic and early neo-hepatic phases with SBP differences of 20.8 ± 0.8 mmHg and 22.8 ± 0.8 mmHg, respectively, and MAP differences of 10.0 ± 0.4 mmHg and 9.8 ± 0.4 mmHg, respectively. Higher vasopressor infusion doses were strongly associated with greater differences in femoral-radial SBP and MAP measurements (r = 0.69 for vasopressin, 0.68 for norepinephrine, and 0.68 for epinephrine; p < 0.0001) during the anhepatic phase.

Conclusions

Peripheral invasive blood pressure monitoring may result in underestimation of the central blood pressure, as was seen in all phases of liver transplantation. This may lead to excessive vasopressor use with potentially adverse effects. Although the cause for the difference between femoral and radial artery measurements is unclear, increasing vasopressor infusion dosages appears to contribute. Femoral artery blood pressure monitoring allows clinicians to interpret hemodynamic status and administer appropriate vasopressors more accurately.

## Introduction

Invasive blood pressure monitoring is warranted in patients with significant comorbidities and in high-risk surgeries where immediate, continuous monitoring is required. Although debate persists on the ideal monitoring site, the most common cannulation site in clinical practice is the radial artery due to ease of access and low risk of complications [1]. Femoral artery cannulation, the second preferred cannulation site, has been associated with more difficult placement and a higher risk of complications, including infection, distal ischemia, arteriovenous fistula, and pseudoaneurysm [1,2].

Despite the popularity of the radial artery for invasive blood pressure monitoring, some studies demonstrate inconsistencies between peripheral and central arterial blood pressure values in patients with septic shock, hypothermia, and after cardiopulmonary bypass [3-5]. Compared to the radial artery, the femoral artery has been considered more representative of central arterial pressures [6]. Although peripheral systolic arterial blood pressure is typically higher than central aortic systolic pressures with similar mean arterial pressures, there is a reverse central-to-peripheral blood pressure phenomenon in the aforementioned clinical situations [3-8]. For example, in hypotensive septic patients on high-dose vasopressors, the radial systolic and mean artery pressures have been shown to underestimate central pressures, and clinical decisions relying solely on radial pressures can result in undue administration of vasopressors [5].

Due to major hemodynamic shifts and the potential for significant bleeding during liver transplant surgery, continuous invasive arterial blood pressure monitoring is essential. However, the choice of peripheral and/or central arterial access can vary depending on the institution and clinician's preference. Few studies have compared femoral and radial artery blood pressures in patients undergoing liver transplant surgery [6,8]. To our knowledge, there are no studies investigating the differences between continuous minute-to-minute femoral and radial artery measurements during all three phases of liver transplant surgery and the effect of vasopressors on these blood pressure differences. The goal of this study was to compare intraoperative blood pressure differences between the femoral and radial arteries in liver transplant patients and to assess for any correlation between vasopressor infusion dose and femoral-arterial pressure differences.

## Materials and methods

A retrospective study was performed after obtaining Institutional Review Board approval from Loma Linda University Medical Center in compliance with Health Insurance Portability and Accountability Act regulations (IRB# 5200470; Liver Transplant Hemodynamics and Transfusion Practices). Patient demographics, femoral and radial arterial blood pressure data, and vasopressor dosages were obtained from the electronic medical record of patients who underwent liver transplant surgery at our institution between January 2019 and December 2019. Patients who experienced cardiopulmonary arrest during the surgery and patients who had missing blood pressure or clamp time data on the electronic medical record were excluded from the study.

For all patients, the radial artery was cannulated with a standard 20 gauge 1.75-inch radial artery catheter (Arrow, Teleflex, Wayne, Pennsylvania), and the femoral artery was cannulated with a standard 18 Gauge 6.29-inch femoral arterial catheter (Arrow). Two different transducers, set to the level of the patient’s right atrium and zeroed to atmospheric pressure, were used to measure radial and femoral arterial blood pressures. Intraoperative clinical decisions by the anesthesiologist were based on femoral arterial blood pressure with the goal to maintain mean arterial pressure (MAP) > 65 mmHg.

Minute-by-minute blood pressure data (systolic blood pressure (SBP), diastolic blood pressure (DBP), and MAP) and vasopressor infusion dosage were obtained for each patient during the pre-anhepatic, anhepatic, and neo-hepatic phases. The pre-anhepatic phase was defined as 60 minutes prior to clamping the inferior vena cava (IVC). The anhepatic phase was defined as the first 45 minutes after clamping of the IVC. Neo-hepatic phase consisted of the first 60 minutes following the removal of the IVC and portal vein clamps.

For data analysis, the difference between femoral-radial blood pressures (SBP, DBP, and MAP) was measured at each time-point of the pre-defined stages of liver transplantation and averaged for all patients per time-point. This was calculated with a paired t-test using data from the radial arterial blood pressure subtracted from the femoral arterial blood pressure for each blood pressure variable (SBP, DBP, and MAP). For each individual vasopressor (vasopressin, norepinephrine, and epinephrine), the mean infusion dosage was recorded at each time point during the three defined phases of liver transplantation. The correlation between vasopressor infusion dose and the mean difference in blood pressure was subsequently calculated using the Spearman correlation coefficient.

## Results

Patient demographics

A total of 103 patients underwent liver transplantation surgery between January 2019 and December 2019 at Loma Linda University Medical Center. However, 43 patients were excluded from the study due to missing blood pressure data, clamping times, or intraoperative cardiopulmonary arrest. Of the resulting 60 patients included in our study, 37 were male (62%). The mean age of our patients was 54.3 ± 11.3 years (range: 19 to 74 years), the mean body mass index (BMI) was 29.8 ± 5.8 kg/m^2^ (range: 18.5 to 42.5 kg/m^2^), and the mean Model for End-Stage Liver Disease (MELD) score was 30.4 ± 8.1 (range: 15 to 40).

Central and peripheral arterial blood pressure comparisons

The mean difference between femoral-radial SBP, DBP, and MAP measurements of all 60 patients is demonstrated in Table 1.

**Table 1:** Mean difference between femoral and radial arterial blood pressures during all three phases of liver transplant surgery (pre-anhepatic, anhepatic, and neo-hepatic phase). Femoral/radial BP: mean (standard deviation). Mean difference of femoral and radial BP: mean difference (95% confidence interval). P-value: paired t-test. BP: blood pressure; SBP: systolic blood pressure; DBP: diastolic blood pressure; MAP: mean arterial pressure.

	Pre-anhepatic				Anhepatic				Neo-hepatic			
	Femoral BP	Radial BP	Mean difference	P-value	Femoral BP	Radial BP	Mean difference	P-value	Femoral BP	Radial BP	Mean difference	P-value
SBP	120.7 (19.3)	104.3 (21.9)	16.5 (15.8-17.2)	<0.0001	133.5 (23.0)	112.7 (27.4)	20.8 (20.0-21.6)	<0.0001	128.6 (21.6)	105.8 (22.0)	22.8 (22.0-23.7)	<0.0001
DBP	57.8 (15.2)	54.2 (12.3)	3.6 (3.3-3.9)	<0.0001	63.9 (16.6)	59.4 (14.0)	4.5 (4.2-4.9)	<0.0001	54.0 (16.0)	50.7 (13.8)	3.3 (3.0-3.7)	<0.0001
MAP	78.7 (14.7)	70.9 (14.5)	7.9 (7.5-8.2)	<0.0001	87.1 (16.6)	77.2 (17.5)	10.0 (9.6-10.4)	<0.0001	78.9 (15.7)	69.0 (17.6)	9.8 (9.4-10.3)	<0.0001

**Figure 1:**
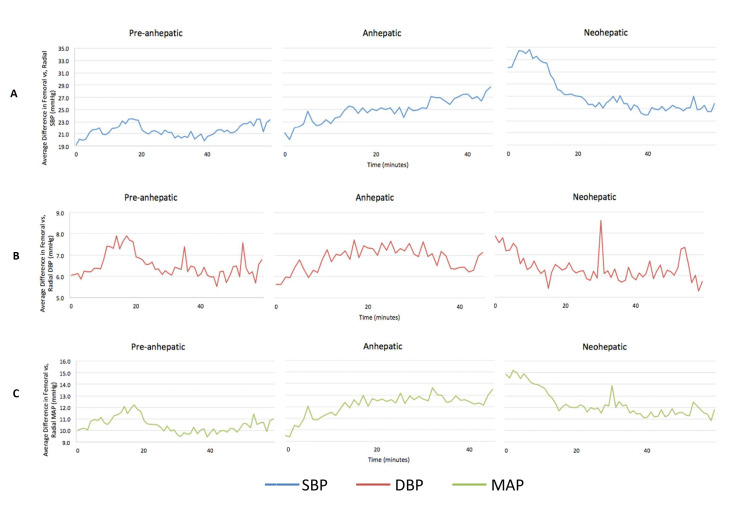
Mean difference between femoral and radial arterial blood pressures during all three phases of liver transplant surgery (pre-anhepatic, anhepatic, and neo-hepatic phase). Figure A demonstrates a statistically significant difference between the mean femoral and radial systolic blood pressure (SBP) (blue) during all three phases of surgery (p < 0.0001), which peaks during the neo-hepatic phase. Figure B shows a statistically significant difference between the diastolic blood pressure (DBP) (red) for femoral and radial arterial lines (p < 0.0001). Lastly, mean arterial pressure (MAP) (green) is shown in Figure C, with a statistically significant difference between femoral and radial arterial MAPs (p < 0.0001).

The mean difference between femoral-radial SBP, DBP, and MAP measurements at each specific time point throughout the operation is visualized in Figure 1.

The mean femoral arterial SBP, DBP, and MAP were all statistically significantly higher than the radial artery measurements during all three phases of liver transplant surgery (p < 0.0001 for SBP, DBP, and MAP) (Table 1 and Figure 1). The mean SBP difference was 16.5 ± 0.7 mmHg, 20.8 ± 0.8 mmHg, and 22.8 ± 0.8 mmHg during the pre-anhepatic, anhepatic, and neo-hepatic phases, respectively (Table 1 and Figure 1A). The mean DBP difference was 3.6 ± 0.3 mmHg, 4.5 ± 0.3 mmHg, and 3.3 ± 0.3 mmHg for the pre-anhepatic, anhepatic, and neo-hepatic phases, respectively (Table 1 and Figure 1B). The mean MAP difference was 7.9 ± 0.4 mmHg, 10.0 ± 0.4 mmHg, and 9.8 ± 0.4 mmHg during the pre-hepatic, anhepatic, and neo-hepatic phases, respectively (Table 1 and Figure 1C). The mean SBP and MAP differences are highest during the late anhepatic and early neo-hepatic phases; this difference decreases shortly after (Figures 1A, 1C).

Vasopressor infusion dosages

**Figure 2:**
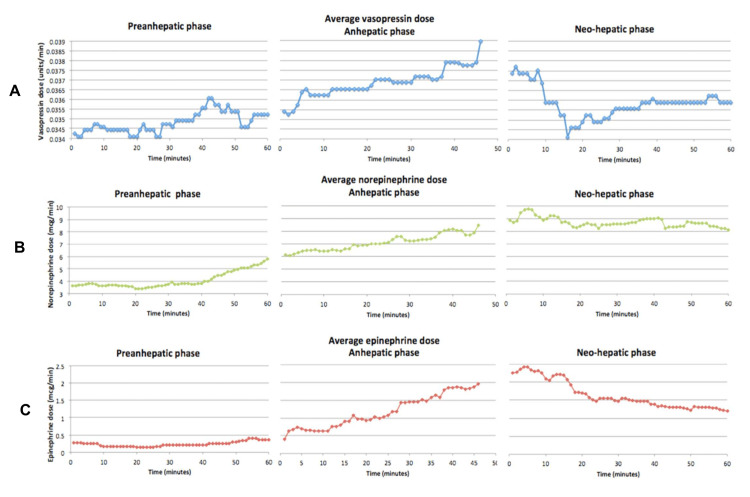
Mean infusion doses of vasopressin (A), norepinephrine (B), and epinephrine (C) during pre-anhepatic, anhepatic, and neo-hepatic stages of liver transplant surgery for all 60 patients.

The average doses of the vasopressor infusions for vasopressin, norepinephrine, and epinephrine were documented for the same time points as the blood pressure values for all 60 patients (Figure 2). No other vasopressor infusions were used for the study patients. Vasopressor infusion doses steadily increased as the surgery progressed, with individual pressors peaking in the anhepatic or neo-hepatic phases, and subsequently decreasing shortly thereafter (Figure 2).

Correlation between vasopressor infusion doses and mean blood pressure differences

Table 2 lists the Spearman correlation coefficients for the vasopressors and mean SBP, DBP, and MAP differences during each phase of liver transplantation.

**Table 2:** Spearman correlation coefficients (R) for the relationship between each vasopressor infusion dose and the blood pressure difference between radial and femoral SBP, DBP, and MAP during all three phases of liver transplant surgery. The p-values are listed below each R-value. SBP: systolic blood pressure; DBP: diastolic blood pressure; MAP: mean arterial pressure; Vaso: vasopressin; Norepi: norepinephrine; Epi: epinephrine.

Pre-anhepatic				Anhepatic				Neo-hepatic			
Spearman correlation coefficients, N = 60 Prob > [r] under H0: Rho = 0				Spearman correlation coefficients, N = 45 Prob > [r] under H0: Rho = 0				Spearman correlation coefficients, N = 60 Prob > [r] under H0: Rho = 0			
	SBP diff.	DBP diff.	MAP diff.		SBP diff.	DBP diff.	MAP diff.		SBP diff.	DBP diff.	MAP diff.
Vaso R	-0.09761	-0.47118	-0.49682	Vaso R	0.88859	0.27314	0.68844	Vaso R	0.06617	0.39556	0.18763
P-value	0.4581	0.0001	<0.0001	P-value	<0.0001	0.0663	<0.0001	P-value	0.6154	0.0018	0.1511
Norepi R	0.14581	-0.47613	-0.27551	Norepi R	0.86498	0.26905	0.67734	Norepi R	0.46736	0.27844	0.45516
P-value	0.2663	0.0001	0.0331	P-value	<0.0001	0.0706	<0.0001	P-value	0.0002	0.0312	0.0003
Epi R	0.08987	-0.54691	-0.19772	Epi R	0.88909	0.24897	0.68271	Epi R	0.85243	0.47423	0.79231
P-value	0.4947	<0.0001	0.1300	P-value	<0.0001	0.0952	<0.0001	P-value	<0.0001	0.0001	<0.0001

Pre-anhepatic Phase

During the pre-anhepatic phase, the correlation between individual vasopressors and the mean SBP difference was not statistically significant (Table 2). Interestingly, there was a statistically significant negative correlation between each vasopressor and the mean DBP difference during the pre-anhepatic phase (R = -0.47 for vasopressin, -0.48 for norepinephrine, and -0.55 for epinephrine) (Table 2). Similarly, there was also a negative correlation between vasopressin and norepinephrine and the MAP difference (R = -0.50 for vasopressin and -0.28 for norepinephrine) (Table 2).

Anhepatic Phase

During the anhepatic phase, there was a strong positive correlation between vasopressor infusion dose and the mean SBP difference (R = 0.89 for vasopressin, 0.86 for norepinephrine, and 0.89 for epinephrine; p < 0.0001) (Table 2). The positive correlation between vasopressor infusion dosage and the mean MAP difference was also statistically significant (R = 0.69 for vasopressin, 0.68 for norepinephrine, and 0.68 for epinephrine; p < 0.0001) (Table 2). The Spearman correlation coefficient for the vasopressors and the mean DBP difference was not statistically significant (Table 2).

Neo-Hepatic Phase

For the neo-hepatic phase, statistically significant positive correlations were found between the norepinephrine and epinephrine doses and the mean SBP difference between femoral-radial artery measurements (R = 0.47 for norepinephrine and 0.85 for epinephrine) (Table 2). Norepinephrine and epinephrine infusions were also positively correlated with the MAP differences (R = 0.46 for norepinephrine and 0.79 for epinephrine). There was a positive correlation between all vasopressor infusions and the mean DBP difference (R = 0.40 for vasopressin, 0.28 for norepinephrine, and 0.47 for epinephrine) (Table 2).

## Discussion

With the prevalence of significant hemodynamic variability in orthotopic liver transplantation, invasive arterial blood pressure monitoring is essential in providing anesthesiologists with data to support clinical decision-making. Reliable and accurate methods for measuring blood pressure are vital to reducing perioperative morbidity and mortality, especially for critically ill patients undergoing high-risk surgery [6]. While the peripheral and central MAPs are similar in healthy patients, the peripheral artery typically has a higher SBP than the central artery due to vascular impedance and resonance resulting in pulse amplification [6]. However, the accuracy of peripheral arterial blood pressure values in estimating the central pressures of critically ill patients has been called into question by many studies [3-9]. In fact, for patients on high-dose vasopressors in shock or weaning off cardiopulmonary bypass, the radial artery has been shown to underestimate central blood pressure [3-5,10]. Compared to the radial artery, the femoral artery has been considered more representative of central arterial pressures [6].

**Figure 3:**
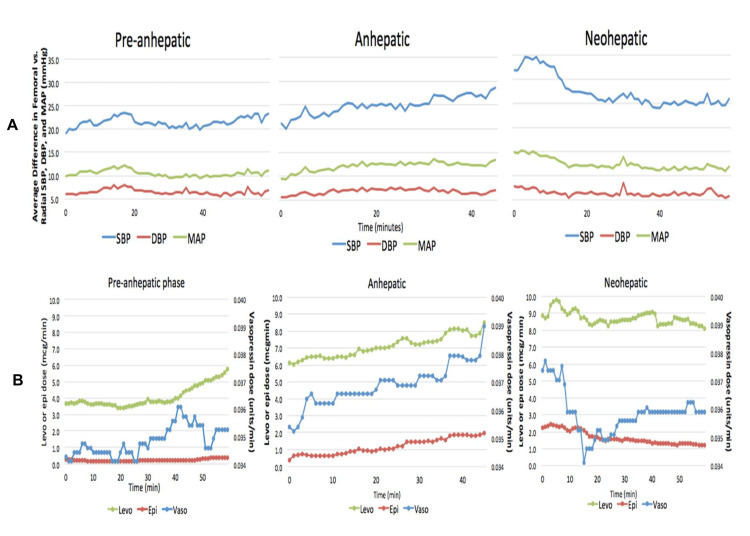
The mean difference between femoral and radial arterial blood pressures during all three phases of liver transplant surgery (pre-anhepatic, anhepatic, and neo-hepatic phase) overlayed on each other (A). Mean infusion doses of vasopressin, norepinephrine, and epinephrine during pre-anhepatic, anhepatic, and neo-hepatic stages of liver transplant surgery overlayed on each other (B). SBP: systolic blood pressure; DBP: diastolic blood pressure; MAP: mean arterial pressure.

With the high volume of liver transplantation surgeries at our institution, our study demonstrates a similar phenomenon in which we appreciate significant blood pressure differences, with femoral artery values consistently measuring higher than the radial artery. This phenomenon is visualized in Figure 1, where the mean femoral SBP, DBP, and MAP were all higher than the radial artery values. This difference continues to widen as the liver transplant surgery progresses from the pre-anhepatic phase to the anhepatic phase, peaking in the late anhepatic stage or the early neo-hepatic stage (Figure 1). This is most prominent in the SBP difference right after reperfusion with a mean SBP difference of 22.8 mmHg. The increased difference between peripheral and central blood pressures tends to follow increased doses of vasopressors (Figure 3).

Few articles compare BP measurement differences specifically in liver transplant patients and the results are conflicting (Table 3).

**Table 3:** A literature review of studies comparing femoral and radial arterial blood pressure differences in orthotopic liver transplantation. SBP: systolic blood pressure; DBP: diastolic blood pressure; MAP: mean arterial pressure; IVC: inferior vena cava.

	Number of patients	Type of study	Time points	SBP difference (mmHg)	DBP difference (mmHg)	MAP difference (mmHg)
Arnal et al. [7]	72	Observational	7 time points: baseline, 10 minutes before IVC clamp, 10 minutes after IVC clamp, 10 minutes before reperfusion, within 3 minutes of reperfusion, 10 minutes after reperfusion, end of biliary reconstruction	Statistical significance during reperfusion (16 mmHg)	N/A	No significant difference throughout the study
Acosta et al. [9]	47	Observational	5 time points: the beginning of surgery, before reperfusion, when MAP reached the lowest value in the first 5 minutes of reperfusion, 5 minutes after the start of reperfusion, end of surgery	N/A	N/A	No significant differences throughout the surgery
Lee et al. [10]	25	Prospective observational	4 time points: 30 minutes after induction, 30 minutes after the start of the anhepatic phase, 30 minutes after graft reperfusion, 30 minutes after bile duct anastomosis	14.9 ± 24.8 (p < 0.001) overall, the difference started after portal vein clamping	Clinically acceptable agreement at all time points	4.8 ± 4.5 (p < 0.001), clinically acceptable agreement at all time points
Our study (Kim et al.)	60	Retrospective observational	165 time points: 1-minute intervals of 60 minutes prior to anhepatic, 1-minute intervals of 45 minutes of anhepatic, 1-minute intervals of 60 minutes after reperfusion	Pre-anhepatic: 16.5 ± 0.7; anhepatic: 20.8 ± 0.8; neo-hepatic: 22.8 ± 0.8 (p < 0.0001)	Pre-anhepatic: 3.6 ± 0.3; anhepatic: 4.5 ± 0.3; neo-hepatic: 3.3 ± 0.3 (p < 0.0001)	Pre-anhepatic: 7.9 ± 0.4; anhepatic: 10.0 ± 0.4; neo-hepatic: 9.8 ± 0.4 (p < 0.0001)

Arnal et al. [7] observed statistically significant differences in femoral and radial SBP of 16 mmHg during reperfusion with no significant MAP differences, using seven time points near transitions between each liver transplant phase. Acosta et al. [9] found no clinical or statistical significance when comparing femoral and radial artery MAP values at five time points in liver transplant patients. Another study by Lee et al. [10] found clinically significant differences between femoral and radial SBPs of 14.9 mmHg following clamping of the portal vein and continuing for the remainder of the surgery; however, the MAPs were not clinically significantly different.

In contrast, our study found statistically significant differences between the femoral and radial mean SBP, DBP, and MAP during all phases of liver transplantation surgery (p < 0.0001). A possible explanation for the difference in our findings is that our study examined continuous minute to minute time-points and a larger number of total time points (60 minutes for pre-anhepatic, 60 for neo-hepatic, and 45 for anhepatic phase), providing a more comprehensive picture of blood pressure measurements throughout the operation. Similar to the study by Arnal et al. [7], our widest femoral to radial mean SBP difference also occurred during the neo-hepatic phase.

The mechanisms responsible for the difference in femoral and radial artery blood pressure measurements in liver transplant surgery are not well understood. One theory is that increasing vasopressors constrict smaller caliber vessels more and reduce flow to the radial artery, contributing to the difference seen between radial and femoral artery measurements. Another theory proposes that extreme peripheral vasodilation, as in the early neo-hepatic phase of liver transplantation, can result in decreased distal pressures compared to central pressures, similar to that seen in post-cardiopulmonary bypass physiology [8]. Other possible explanations include but are not limited to pulse wave reflection, the diameter of the artery, microcirculation anatomy, and inflammatory mediators [6,7].

A recent, retrospective analysis of liver transplant surgery revealed patients had an increased risk for 30-day mortality with sustained intraoperative MAPs below 60 [11]. Although hypotension is to be avoided, it is equally important to avoid treating hypotension too aggressively in this patient population as they often have decreased systemic vascular resistance and a lower baseline MAP. In critically ill patients, making clinical decisions solely based on peripheral artery measurements can lead to unnecessary vasopressor administration, with the potential negative side effects of organ ischemia, hyperglycemia, tachycardia, cardiac arrhythmias, and increased metabolic demand and oxygen consumption [5].

Small studies in liver transplant surgery, cardiac surgery, and the intensive care unit argue that utilizing peripheral invasive blood pressure monitoring alone may result in underestimating the central blood pressure [3-12]. Our study found a mean SBP difference as high as 22.8 ± 0.8 mmHg during the neo-hepatic phase and relying exclusively on the peripheral pressures, in this case, presents a considerably different clinical picture for the provider. Central blood pressure monitoring may help to avoid complications associated with vasopressor use.

Some studies suggest that high-dose vasopressors contribute to the discrepancy between central and peripheral blood pressure, creating a cycle that could lead to significant morbidity and mortality [5]. Our study demonstrates a statistically significant positive correlation between vasopressor use and blood pressure differences between femoral and radial arteries, particularly during the anhepatic and neo-hepatic phase, during which higher doses of vasopressors are typically needed and contribute to wider differences between central and peripheral blood pressure. As hemodynamics stabilize following reperfusion, blood pressure differences decrease with decreased vasopressor use (Figure 3). In the pre-anhepatic phase, patients are typically on low-dose vasopressors with smaller differences in peripheral and central blood pressure.

Limitations

A limitation of our study was that vasopressor boluses were not included in our data, and only vasopressor infusion doses were included in the data analysis. Because all our patients were on vasopressor infusions at the time points measured, we did not compare the effect of vasopressors to no vasopressors on the blood pressure difference. Additionally, our study did not account for other confounding variables, such as the physiologic effects of liver failure or reperfusion on peripheral vs. central artery vascular changes.

Though our pilot study shows statistically significant differences between femoral and radial blood pressure measurements, it is difficult to quantify these data into clinical significance. To determine clinical significance, a future study would compare two groups of patients managed separately via femoral or radial blood pressure readings to detect if differences exist in the vasopressors utilized and outcomes of morbidity and mortality.

## Conclusions

To our knowledge, our pilot study is the first to examine minute-to-minute blood pressure differences in all three phases of liver transplant surgery, with a robust dataset examining femoral and radial arterial measurements. Based on the results, central blood pressure monitoring is recommended in liver transplant surgeries and major surgeries with hemodynamic shifts, massive blood loss, and multiple vasopressor usages. Multiple vasopressors are a contributing factor to the differences between central and peripheral blood pressure measurements. Central arterial blood pressure measurements allow for a more accurate interpretation of hemodynamic status and the appropriate management of vasopressors. Further studies examining additional factors such as blood pH, temperature, patient age, MELD score, and type of operation that may cause the significant differences between central and peripheral arterial blood pressure measurements may yield further insight into how the vasculature reacts in the perioperative period.
